# Habitat‐Dependent Provisioning Patterns Are Modulated by Weather Conditions in a Rapidly Declining Farmland Raptor

**DOI:** 10.1002/ece3.72969

**Published:** 2026-01-19

**Authors:** S. Sangeeth Sailas, Matthias Tschumi, Martin U. Grüebler, Filip Reipricht, Pascal Stroeken, Ronald van Harxen, Martin Šálek

**Affiliations:** ^1^ Department of Zoology, Faculty of Science University of South Bohemia České Budějovice Czechia; ^2^ Swiss Ornithological Institute Sempach Switzerland; ^3^ Raptor Protection of Slovakia Bratislava Slovakia; ^4^ STONE Aalten the Netherlands; ^5^ Czech Academy of Sciences Institute of Vertebrate Biology Brno Czechia; ^6^ Faculty of Environmental Sciences Czech University of Life Sciences Prague Prague‐Suchdol Czechia

**Keywords:** agricultural intensification, food limitation, habitat composition, Little Owl, prey composition, provisioning rate

## Abstract

Agricultural intensification and concomitant reduction in high‐quality habitats represent one of the major threats to farmland biodiversity in Europe. The Little Owl *Athene noctua* is an avian farmland predator whose population rapidly declined across many European countries. Food limitation during the breeding season has been considered a key factor driving this decline. However, it remains unclear how the quality of agricultural habitats affects parental food provisioning across different Little Owl populations. In addition, little is known about how weather conditions may modulate provisioning patterns dependent on habitat quality. Using nestbox cameras, we monitored parental food provisioning of Little Owls in four European countries with contrasting farmland structure (Czechia, Slovakia, Germany, Netherlands), covering 40 nests and 58 broods during the period 2002–2022. In particular, we investigated the interacting effects of habitat quality within Little Owl territories and weather conditions on parental food provisioning and nestling diet. Across all countries, provisioning rate and biomass increased with the area of high‐quality habitats, showed a quadratic relationship with temperature, and decreased with wind speed. Additionally, high‐quality habitats, compared with low‐quality habitats, acted as a buffer against adverse weather, allowing parents to provision more biomass at low temperatures and increase provisioning rates at high wind speeds. The changes in prey composition due to poor weather conditions differed considerably between habitats of high and low quality, suggesting that under poor conditions, Little Owls are able to access more insects and voles in high‐ than low‐quality habitats. Our findings highlight that increasing the area of high‐quality habitats, hand in hand with enhancing habitat heterogeneity within Little Owl territories, should represent the prime conservation activity to reduce the risk of food limitation across contrasting farmlands, particularly under poor weather conditions.

## Graphical abstract

We monitored parental food provisioning of Little Owls in four European countries with contrasting farmland structure using nestbox cameras and demonstrated that across all countries, provisioning rate and biomass increased with the area of high‐quality habitats. Additionally, high‐quality habitats acted as a buffer against adverse weather, allowing parents to provision more biomass at low temperatures and increase provisioning rates at high wind speeds. The changes in prey composition due to poor weather conditions differed considerably between habitats of high and low quality, suggesting that under poor conditions, Little Owls are able to access more insects and voles in high than in low‐quality habitats.

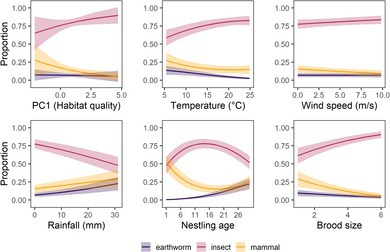

## 1 Introduction

Habitat quality is a key factor influencing individual survival and reproductive success (Newton 1991; Johnson et al. 2006). Individuals breeding in low‐quality habitats with patchy or reduced prey availability suffer increased fitness costs compared to individuals breeding in high‐quality habitats (Olsson et al. 2002; Fuller 2012; Sumasgutner et al. 2014). For example, in altricial birds, breeding in low‐quality habitats forces parents to expend more energy on foraging, yet still results in reduced food provisioning to the nest (Catry et al. 2013). Higher foraging effort can increase physiological costs and put adults at higher risk of predation (Lima 2009; Fowler and Williams 2017), while reduced amounts of food result in reduced body condition of offspring (Garrett et al. 2022a). Together, low‐quality habitats therefore lead to reduced adult and offspring survival at the individual level (Olsson et al. 2002; Tremblay et al. 2005; Michel et al. 2022) and consequently, reduced productivity, survival rates and overall viability of the population (Donald and Forrest 1995; Fuller 2012; Demerdzhiev et al. 2022).

Since the second half of the last century, landscape structure and management intensity in European agricultural landscapes have drastically changed as a result of agricultural intensification (Kehoe et al. 2017). This resulted in large‐scale landscape homogenisation and a loss and degradation of high‐quality semi‐natural habitats with high prey availability (Emmerson et al. 2016; Jeliazkov et al. 2016; Šálek et al. 2018, 2021). This drastically eroded the quantity and quality of foraging, nesting, and roosting habitats for species that had earlier adapted to and thrived under low‐intensity small‐scale farming (Buckingham et al. 2006; Grande et al. 2018; Šálek, Kadava, et al. 2025; Šálek et al. 2026). Unsurprisingly, populations of farmland bird species have severely declined, with the most profound population reductions happening in the regions with strongest farmland intensification (Donald et al. 2001; Reif et al. 2008; Gómez‐Catasús et al. 2025).

The Little Owl *Athene noctua* inhabits a variety of natural and human‐modified open and semi‐open habitats (Van Nieuwenhuyse et al. 2023), though it is largely associated with agricultural landscapes in Central and Western Europe (Fattebert et al. 2018; Grzywaczewski 2009; Šálek et al. 2016). Here, its distribution depends on high‐quality foraging habitats, such as grasslands, gardens, hedges, and orchards (Šálek and Lövy 2012; Apolloni et al. 2018; Van Nieuwenhuyse et al. 2023). While populations have decreased since the 1950s and many still continue to decline, some western European populations have stabilised and some are even increasing (Šálek and Schröpfer 2008; Le Gouar et al. 2011; Chrenková et al. 2017; Van Nieuwenhuyse et al. 2023). Food limitation during the nesting period is considered the main driver of population declines (Thorup et al. 2010; Perrig et al. 2014; Van Nieuwenhuyse et al. 2023). For example, Grüebler et al. (2018) demonstrated that Little Owl parents occupying low‐quality territories provisioned only 63% of the biomass compared to parents in high‐quality territories (see also Jacobsen et al. 2016). Although this highlights the importance of high‐quality habitats for parental provisioning in Little Owls, responses to habitat effects can vary across populations (Zeigler et al. 2021; Hasui et al. 2024). Hence, the generalisability of this relationship, as well as the mechanism by which such adjustments in provisioning occur, remain not yet unequivocally demonstrated.

Along with habitat quality, weather conditions can affect parental provisioning, in terms of numbers, biomass, and composition of provisioned prey (Wiley and Ridley 2016; Garcia‐Heras et al. 2017). Adverse weather such as low temperatures, heavy rainfall, and strong wind may significantly reduce prey availability and foraging efficiency (White 2008; Fisher et al. 2015; Garrett et al. 2022b) and are also identified as important drivers of habitat selection and survival in Little Owls (Sunde et al. 2014; Perrig et al. 2024). Moreover, at low temperatures, nestlings may require additional food to compensate for higher thermoregulatory costs (Winkler et al. 2013). Habitat quality may further mediate the effects of adverse weather. If the effects are additive, adverse weather conditions will reduce food provisioning irrespective of habitat quality. In contrast, if the effects are interactive, high‐quality habitats may buffer against the negative effects of adverse weather by offering alternative prey or foraging sites. While some studies have examined how high‐quality habitats buffer the negative impact of harsh weather conditions on breeding success, population size, and stress physiology of birds, their role in shaping provisioning behaviour and diet composition remains largely unexplored (Newson et al. 2014; Flesch et al. 2015; Cīrule et al. 2017).

To fill these knowledge gaps, we investigated the effect of habitat quality and weather conditions on parental provisioning patterns and prey composition of breeding Little Owls across four European countries (Czechia, Slovakia, Germany, and the Netherlands). We hypothesised that the positive effect of habitat quality on parental provisioning patterns, in terms of provisioning rates (PR) and provisioned biomass (BM), will be consistent across populations. In addition, we expected that adverse weather conditions, such as rainfall and strong wind, would reduce PR and BM by lowering foraging efficiency. We also anticipated a quadratic effect of temperature on provisioning patterns since we expected prey availability to be reduced at extreme temperatures. Finally, we expected interactive effects of weather conditions and habitat quality, such that high‐quality habitats would buffer against the effects of adverse weather conditions. Altogether, our research aims to shed light on the factors determining parental provisioning in Little Owls, particularly the interplay between habitat quality and weather. These insights will be particularly important in the context of continued farmland habitat degradation and rising frequency of extreme and adverse weather events under ongoing climate change.

## 2 Material and Methods

### 2.1 Study Species and Study Areas

The Little Owl is a small, monogamous, and territorial owl species with high fidelity to occupied territories (Michel et al. 2017; Van Nieuwenhuyse et al. 2023). Although considered a dietary generalist, earthworms, arthropods, birds, and small mammals form the main prey, especially during the nesting period (Šálek, Riegert, and Křivan 2010; Van Nieuwenhuyse et al. 2023; Sailas et al. 2024). The species breeds in cavities of both natural and anthropogenic origin, and clutch sizes usually range from 1 to 7 (Chrenková et al. 2017; Van Nieuwenhuyse et al. 2023). The female incubates the eggs for an average of 28 days, during which the male provisions her with food (Van Nieuwenhuyse et al. 2023). Once the nestlings hatch, the male continues to provision the brooding female and offspring for another 5–7 days, after which the female also participates in provisioning (Grüebler et al. 2018; Van Nieuwenhuyse et al. 2023). Fledging of the chicks occurs 28–32 days after hatching (Van Nieuwenhuyse et al. 2023). Due to dramatic reductions of natural nesting sites in many populations (Van Nieuwenhuyse et al. 2023), conservation activities often focus on large‐scale provisioning of artificial nestboxes (Gottschalk et al. 2011; Van Nieuwenhuyse et al. 2023).

**FIGURE 1:**
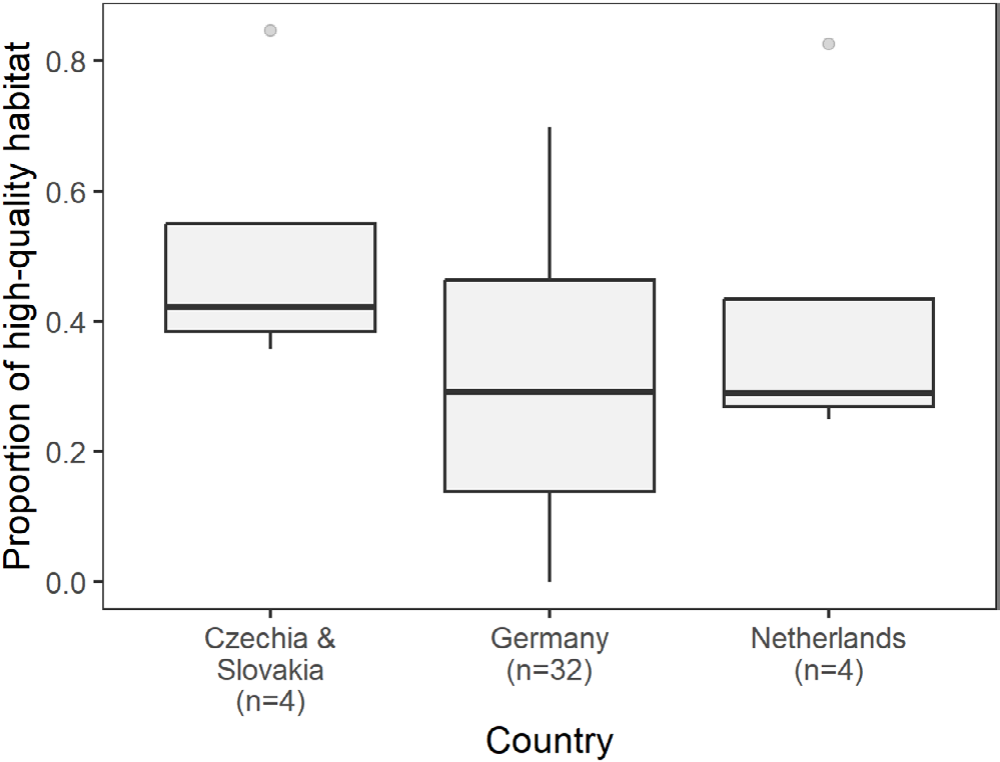
The proportion of high‐quality habitat (i.e., grasslands, gardens, hedges and orchards) in Little Owl territories (i.e., within a 220 m radius around occupied nestboxes) in the studied countries. Czechia and Slovakia were pooled since there was only one territory monitored in Slovakia. The higher variability in the proportion of high‐quality habitat in Germany is partly explained by the larger sample size compared to other countries (see also Table 1).

The study was conducted in four European countries with differing agricultural landscape structure: Czechia, Slovakia, Germany, and the Netherlands (Supplementary Figure S1). In Czechia (Northern and Central Bohemia, 50°25′N, 14°04′E) and Slovakia (Trnava region, 48°19′N, 17°35′E), Little Owls breed within medium‐sized human settlements (villages), including village outskirts or livestock farmsteads (see also Šálek 2014; Sailas et al. 2024). Here, the studied territories (i.e., 220 m radius buffer around Little Owl nestboxes; see also Šálek et al. 2016) were composed of a higher proportion of high‐quality habitats (i.e., grasslands, gardens, hedges, and orchards) compared to other populations (Figure 1), while the wider landscape is dominated by large and homogeneous arable fields. In contrast, Little Owls in the study areas in Germany (Ludwigsburg, Baden‐Württemberg, 48°53′N, 9°11′E) and the Netherlands (GPS: Dongen—51°37′N, 4°54′E, Winterswijk—51°58′N, 6°43′E) breed outside human settlements with a stronghold in traditional fruit orchards (Grüebler et al. 2018) or small family farms (van Harxen et al. 2023; Sailas et al. 2024). Compared to the study areas in Czechia and Slovakia, the surrounding agricultural landscapes in the study areas in Germany and the Netherlands are more heterogeneous and arable fields are interspersed with traditional orchards and grasslands.

### 2.2 Study Design and Nestbox Monitoring

During the period 2002–2022, a total of 58 broods from 40 nestboxes were monitored across the breeding season. Detailed information about the number of monitored nestboxes, broods, years, and camera types in individual countries are provided in Table 1. All studied broods were located in artificial nestboxes and were monitored using nestbox video cameras (Czechia, Slovakia, and the Netherlands) or camera traps (Germany). Brood size was determined either from the cameras (Czechia, Slovakia, Netherlands) or during nestbox visits for ringing of the nestlings (Germany). Nestling age was calculated from the hatching date, observed from the nestbox cameras in Czechia, Slovakia, and the Netherlands and visually estimated using illustrations of Little Owl's chick development from Van Nieuwenhuyse et al. (2023) in Germany. Monitoring periods differed between countries, with broods in Czechia, Slovakia, and the Netherlands being monitored from egg laying up to fledging (up to 44 days after hatching) and broods in Germany monitored only between days 1 and 30 after hatching. We thus restricted our analyses to the shared monitoring period of days 1–30 after hatching in all countries.

**TABLE 1:** Number of nestboxes, broods, monitoring years, provisioned prey items, and camera placement used for the assessment of parental provisioning of Little Owls in each country.

Country	Number of nestboxes	Number of broods	Years monitored	Number of provisioned prey items	Camera placement
Czechia	3	4	2019, 2021–2022	5691	Two cameras were used. One recorded the nestbox interior and the other recorded the entrance from inside
Germany	32	38	2011–2012	6092	One camera trap recording photos was positioned facing the entrance of the nestbox from outside
Slovakia	1	1	2021	1169	One camera recorded the nestbox interior
Netherlands	4	15	2002–2020	30,055	Three cameras were used. One recorded the nestbox interior, and two recorded the entrance from inside and outside the nestbox, respectively

### 2.3 Parental Provisioning

We quantified the provisioning rates per day and identified the provisioned prey from camera recordings. Provisioned prey were categorised into five main classes: mammals, birds, insects, earthworms, and unidentified (see also Sailas et al. 2024). To obtain the daily biomass provisioned, we multiplied the number of prey items of each class by their mean fresh weight obtained from Little Owl provisioning literature (Grüebler et al. 2018; Sailas et al. 2024): mammals = 25 g, birds = 33 g, insects = 0.9 g, earthworms = 3.7 g, unidentified prey = 2.9 g (weighted average of prey weight from the four main categories). However, we acknowledge that this method of calculating daily biomass provisioned does not consider the within‐class variation in prey weight, which for Little Owls can range from 7 to 45 g for mammals, 0.3–2 g for insects and 17–95 g for birds (Grüebler et al. 2018; Sailas et al. 2024).

### 2.4 Habitat Quality and Weather Parameters

To evaluate the effect of habitat quality on provisioning patterns, we first calculated the area of five main habitat types (i.e., grasslands, gardens, orchards, hedges, and arable fields) within Little Owl home ranges (15 ha, corresponding to a 220 m buffer around individual nestboxes) using manually digitised, high‐resolution orthophotos maps (Google Satellite imagery accessed through the HCMGIS plugin) in the software QGIS (QGIS Development Team 2022). We selected an area of 15 ha since it corresponds to the average home range size of Little Owls during the nesting period in Central and Western Europe (Šálek et al. 2016; Šálek, Monoki, et al. 2025). Grasslands included meadows, pastures, and lawns. While gardens and orchards both comprised groups of trees with grassy understorey and were thus structurally similar, gardens were restricted to human settlements and sometimes contained small cultivated patches. Hedges consisted of linear strips of trees or shrubs, often occurring along field margins or road verges. We then classified these habitat types into high‐quality habitats (grasslands, gardens, orchards, hedges) and low‐quality habitats (arable fields) based on the availability and abundance of food resources (for more details see Šálek and Lövy 2012; Grüebler et al. 2018; Van Nieuwenhuyse et al. 2023). In addition, we performed a principal component analysis (PCA) using the area of the five habitat types. The areas were centred and scaled prior to their inclusion in the PCA, which was implemented using the *princomp* function from the *factoextra* package in R (Kassambara and Mundt 2020; R Core Team 2024). The first principal component (PC1) explained 46.1% of the variance and was negatively correlated with gardens, grasslands, and hedges, and positively correlated with arable land. PC1 thus represented a gradient of habitat quality from low‐quality habitats with large areas of arable land to high‐quality habitats with large areas of grasslands, gardens, and hedges (see Table S1 and Figure S2 for more details). The second principal component (PC2) explained 25.1% of the variance and was negatively correlated with orchards and positively correlated with arable land, representing a gradient from orchard‐dominated habitats to fully open habitats (large areas of arable land). For each nestbox, we extracted the scores from the first and second principal components and reversed the signs of the PC scores so that positive values of PC1 indicated high‐quality habitats and positive values of PC2 indicated orchard habitats.

To investigate the effect of weather conditions, we obtained data on average daily temperature (°C), total daily rainfall (mm), and average daily wind speed (m/s) from the nearest meteorological stations for each study area. We accessed the weather data for the Czech study area from https://www.chmi.cz/, for the Slovak study area from https://en.tutiempo.net/ and https://www.visualcrossing.com/, for the German study area from https://opendata.dwd.de/ and for the Dutch study area from https://dataplatform.knmi.nl/. The average distance from weather stations to Czech nestboxes was 25 km, to the Slovak nestbox 26 km, to the German nestboxes 16 km and to the Dutch nestboxes 12 km.

### 2.5 Data Analyses

We used linear mixed‐effects models (LMMs) to evaluate the factors associated with provisioning rate (per day and per chick) and provisioned biomass (per day and per chick). Specifically, the total number (for provisioning rate) and biomass (for provisioned biomass) of provisioned prey for each day and each brood, divided by the number of chicks, were treated as one data point in the analyses. To analyse factors associated with prey composition, we employed a multinomial model. Here, the response variable was a matrix of counts of provisioned prey from the three main categories with the majority of data (insect, mammal and earthworm), with each data point representing a single day from each brood. In both model types, fixed predictor variables included PC1 (habitat quality) and PC2 (orchards), as well as all weather parameters (the linear and quadratic term of temperature, and the linear terms of rainfall and wind speed). In addition, nestling age (as a linear and quadratic term) and brood size were included as fixed effects to account for the variation due to brood characteristics. Brood id nested within nest id was included as a random effect to account for repeated observations from the same brood and nest. Prior to model fitting, we checked for correlations among the predictors using the Pearson's correlation coefficient and confirmed that none were highly correlated (*r* ≥ 0.6).

Model fit was assessed by inspecting residual and QQ‐plots. Upon observing heteroscedasticity in the residuals of the models of provisioning rates and biomass, we applied a cubic‐root transformation to those response variables. All predictors were centred and scaled prior to their inclusion in the models. We implemented the LMMs using the *lmer* function from the *lme4* package (Bates et al. 2015; R Core Team 2024) and the multinomial model using the *mblogit* function in the *mclogit* package in R (Elff 2022; R Core Team 2024). As the multinomial model including brood id nested within nest id as random effects failed to converge, only brood id was retained. However, this also implies that the *p*‐values from the multinomial model may be overly optimistic and should be treated with caution. Model predictions were generated using the *ggeffects* (Lüdecke, Bartel, et al. 2025), and figures were created and arranged using the *ggplot2*, *sjPlot*, and *patchwork* packages (Lüdecke, Aust, et al. 2025; Pedersen 2025; Wickham et al. 2025).

Because PC1 values (high‐quality habitats around nestboxes) differed substantially among countries, we were unable to include country as a predictor in the models. Therefore, the models did not account for potential country‐level differences even if the equipment used for monitoring nestboxes could not be standardised among countries since the research was performed independently by the respective research groups. To test for potential differences between countries, we thus implemented a model with a subset of the data for which the PC1 values overlapped across countries and included country as a fixed effect (Table S2). In addition, for 13% of monitored days in Germany (74 days; 8% of the entire dataset), camera‐traps were active for only half of the day. To account for this, provisioning rates and provisioned biomass of these half‐days were doubled. To check whether the inclusion of these half‐days affected the results, we additionally ran a model excluding these observations (Table S3).

## 3 Results

In total, we recorded 43,007 prey items provisioned to nestlings across 58 broods from 40 nests in all four countries. On average, Little Owls provisioned 15.1 ± 15.5 (mean ± SD) prey items per chick and day. The average biomass provisioned per chick and day was 44 ± 34.9 g (mean ± SD). Insects formed the majority of the diet, accounting for 59.8% of all provisioned prey items, followed by earthworms (15.2%), mammals (4%), and birds (0.5%). The remaining 20.4% consisted of unidentified prey.

### 3.1 Provisioning Rate

**FIGURE 2:**
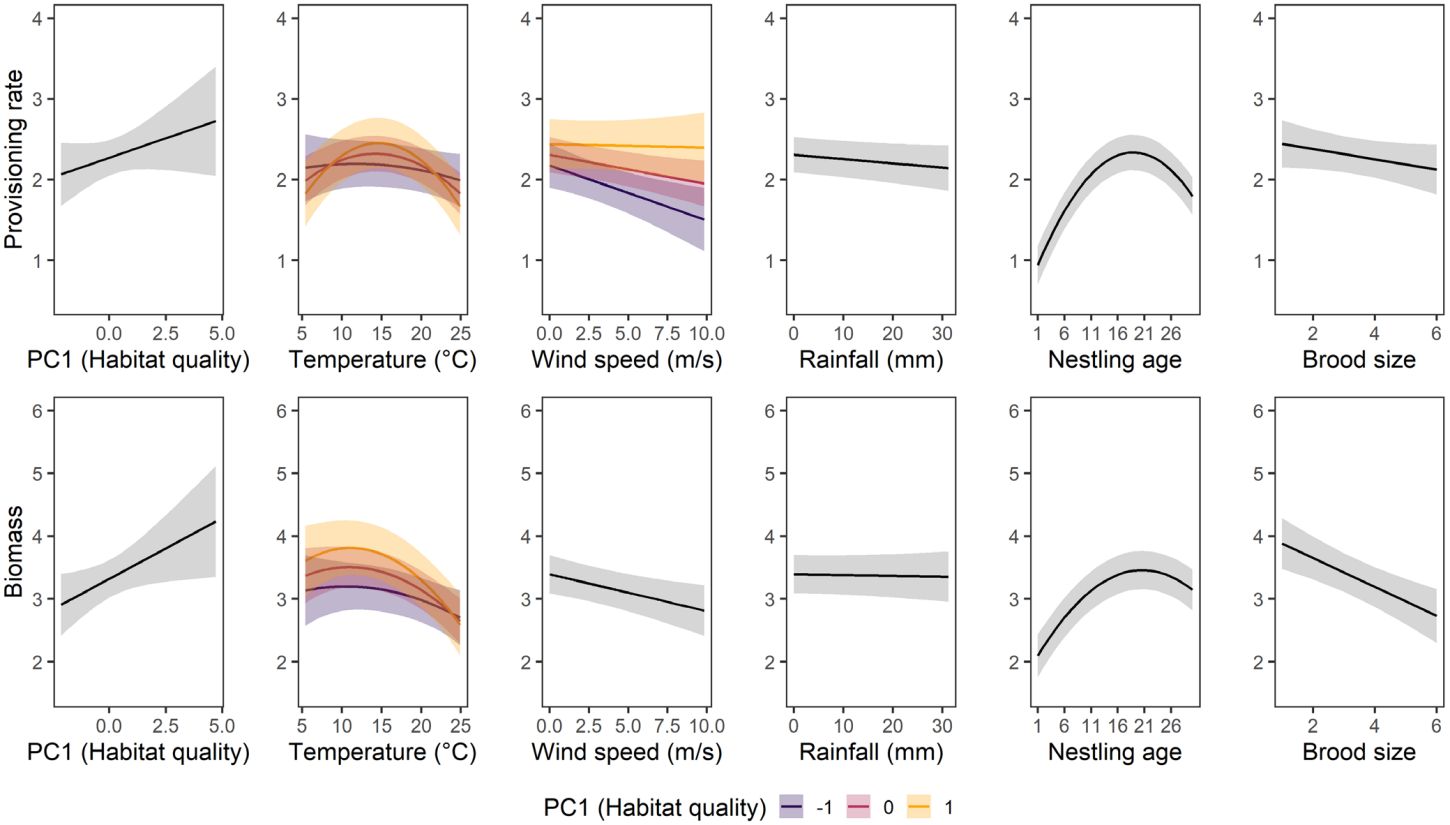
Predicted provisioning rate (per day and chick, upper panel) and provisioned biomass (per day and chick, lower panel) of breeding Little Owls in relation to habitat quality (PC1), weather variables and brood characteristics. Lines depict means and shaded areas 95% confidence intervals. All response variables were cubic‐root transformed and predictors were centred and scaled, though predictors were back‐transformed to plot them in their original scale. Interactive effects are plotted for three values for PC1 (mean and mean ± 1 SD).

The model assessing differences between countries, based on a limited dataset of overlapping PC1 values, indicated no relevant effect of country on provisioning rates (Table S2). The model including the full dataset showed a quadratic relationship of provisioning rate (per chick, PR) with nestling age, increasing from the first day after hatching and decreasing again beyond day 20 after hatching. Although PR tended to be lower in large broods, this association was not significant (Figure 2, Table 2). PR also increased significantly with PC1, whereas no significant relationship was recorded with PC2 (Figure 2, Table 2). Additionally, PR exhibited a significant quadratic relationship with temperature, peaking at intermediate temperatures (13°C–15°C). This effect interacted with PC1, with higher PR at intermediate temperatures at high PC1 values, whereas PR was largely unaffected by temperature at low PC1 values (Figure 2, Table 2). PR also decreased with wind speed with an interaction with PC1 that approached significance, showing that PR decreased with wind speed at low PC1 values but remained stable at high PC1 values. PR was not affected by rainfall (Figure 2, Table 2). Excluding half‐day observations from the dataset did not qualitatively change these results (Table S3).

**TABLE 2:** Model estimates, standard errors (SE) and *p*‐values from the LMMs assessing factors associated with provisioning rate and provisioned biomass for breeding Little Owls. Response variables were cubic‐root transformed, and all predictors were centred and scaled prior to their inclusion in the models. *p* values of statistically significant effects (< 0.05) are given in bold.

Predictors	Provisioning rate			Biomass provisioned		
	Estimates	SE	*p*	Estimates	SE	*p*
(Intercept)	2.19	0.10	**< 0.001**	3.22	0.14	**< 0.001**
Age	0.18	0.02	**< 0.001**	0.25	0.02	**< 0.001**
Age^2^	−0.28	0.01	**< 0.001**	−0.23	0.02	**< 0.001**
Brood size	−0.07	0.05	0.130	−0.26	0.07	**< 0.001**
PC1	0.22	0.09	**0.013**	0.27	0.12	**0.033**
PC2	−0.14	0.08	0.078	−0.18	0.11	0.105
Temp	−0.05	0.02	**0.002**	−0.16	0.03	**< 0.001**
Temp^2^	−0.05	0.01	**< 0.001**	−0.05	0.02	**0.001**
Rain	−0.02	0.01	0.084	−0.01	0.02	0.785
Wind	−0.05	0.02	**0.012**	−0.08	0.03	**0.005**
PC1 × temp	−0.02	0.01	0.203	−0.06	0.02	**0.002**
PC1 × temp^2^	−0.04	0.01	**< 0.001**	−0.02	0.01	0.085
PC1 × wind	0.04	0.02	0.056			

### 3.2 Provisioned Biomass

The factors associated with provisioned biomass (BM) were generally similar to those associated with provisioning rate (PR). However, in contrast to PR, BM showed a less pronounced decline at the end of the nestling period and decreased significantly with increasing brood size (Figure 2, Table 2). Similar to PR, BM increased significantly with PC1, but not with PC2 (Figure 2, Table 2). The peak of the quadratic relationship between temperature and BM occurred at lower temperatures (10°C–12°C) than observed for PR (Figure 2, Table 2). Additionally, BM did not show a sharp decline at low temperatures (5°C–10°C) like PR (Figure 2, Table 2). As for PR, the negative association with wind speed was significant, while the effect of rainfall remained non‐significant (Figure 2, Table 2). As for PR, we also found a significant interaction between temperature and PC1 affecting BM (Table 2). At low temperatures, BM was considerably higher at high PC1 values than at low PC1 values, while there was no difference in BM between high and low PC1 values at high temperatures (Figure 2). In contrast to PR, we found no interaction between wind speed and PC1 for BM. There was a strong positive correlation between BM and PR, even though low provisioning rates were associated with larger than average prey and high provisioning rates with smaller than average prey (see Figure S3).

### 3.3 Prey Composition

**FIGURE 3:**
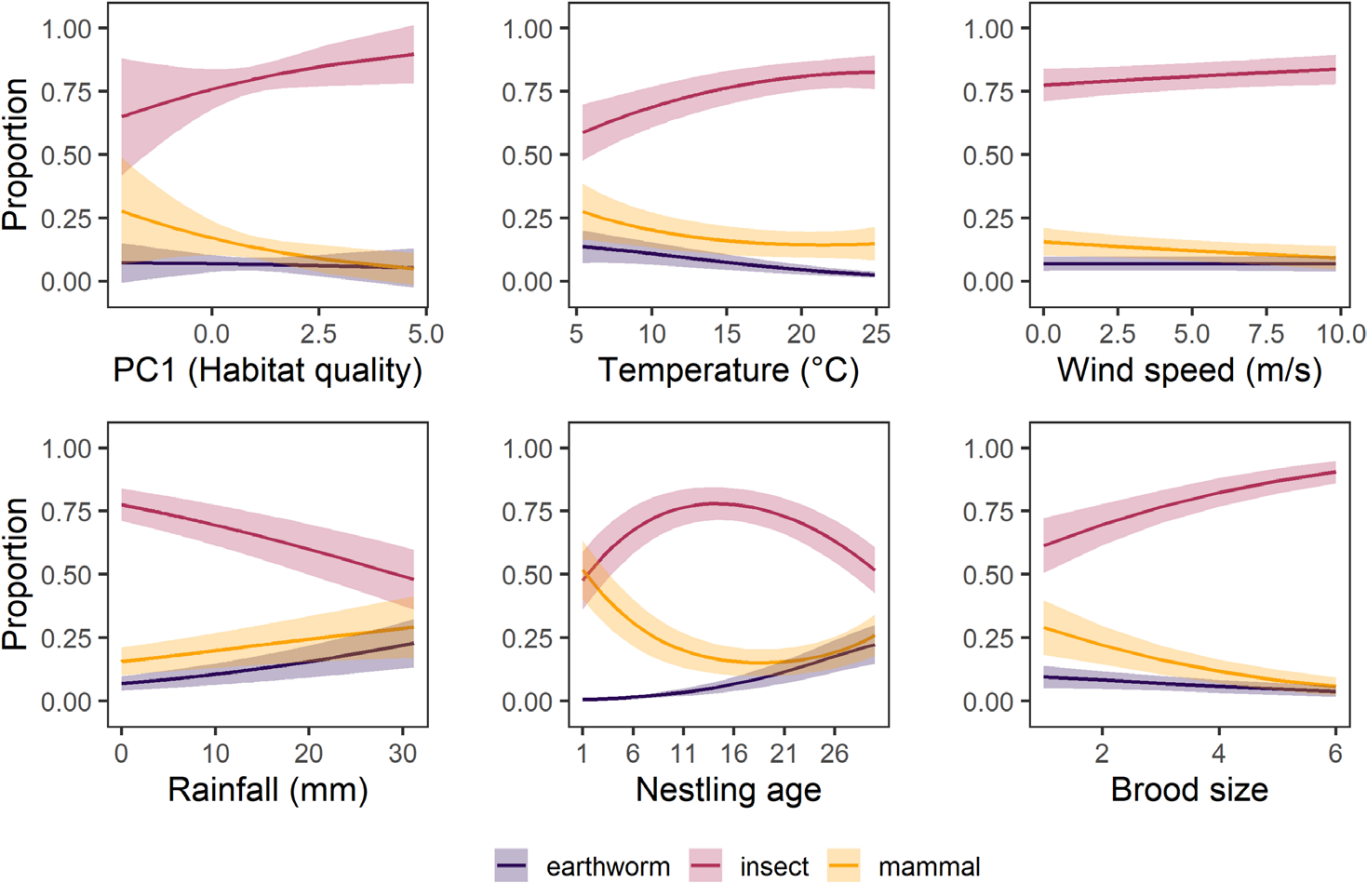
Predicted proportions of main prey types provisioned by breeding Little Owls in relation to habitat quality (PC1), weather variables, and brood characteristics. Lines depict means and shaded areas depict the 95% confidence intervals. All predictors were centred and scaled prior to their inclusion in the model but were back‐transformed to plot them in their original scale.

The proportion of mammals, the prey item with the highest biomass, decreased with brood size and with nestling age (Figure 3, Table S4). The proportion of earthworms increased with nestling age and was slightly reduced in larger broods, whereas the proportion of insects exhibited a quadratic relationship with nestling age, peaking at intermediate ages and increasing with brood size.

The provisioning of mammals decreased and insects increased with PC1, but neither were associated with PC2 (Figure 3). Although the proportion of earthworms tended to decrease with PC1, this relationship was not significant, and no effect was detected for PC2.

Weather parameters also influenced the provisioning of different prey types. High temperatures reduced the proportion of mammals and earthworms but increased that of insects (Figure 3). High wind speeds reduced the provisioning of mammals and increased that of insects, while earthworm provisioning was unaffected (Figure 3). Increasing rainfall resulted in an increased provisioning of mammals and earthworms but decreased the provisioning of insects (Figure 3).

**FIGURE 4:**
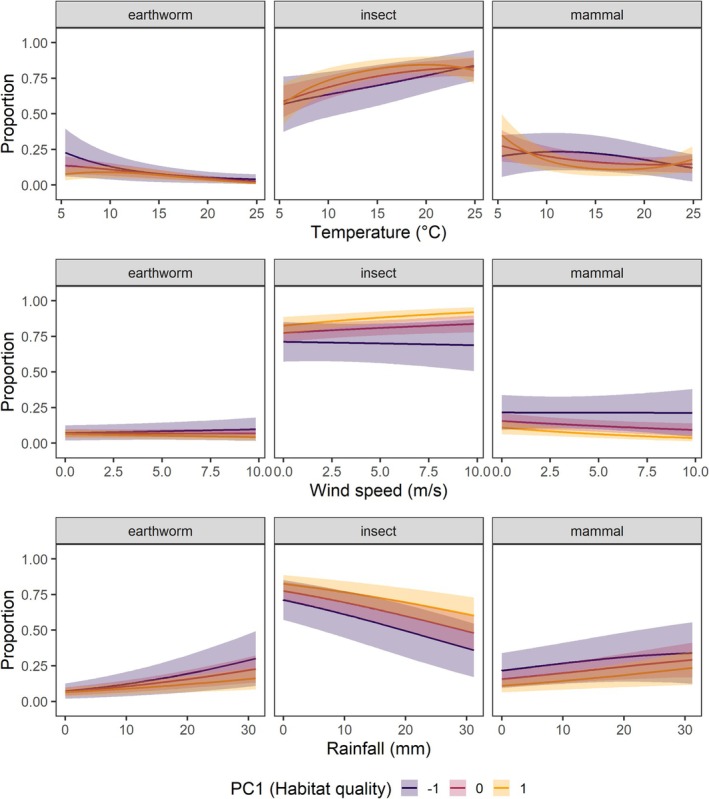
Interactive effects of habitat quality (PC1) and weather conditions. Shown are the predicted proportions of main prey types provisioned by breeding Little Owls in relation to weather variables for three PC1 values (mean and mean ± 1SD). Lines depict means and shaded areas depict the 95% confidence intervals. All predictors were centred and scaled prior to their inclusion in the model but were back‐transformed to plot them in their original scale.

We also found significant interactions between PC1 and weather parameters for the three main prey types. High PC1 values were associated with increased proportions of mammals at low temperatures but decreased proportions at intermediate and high temperatures. In contrast, insects were considerably higher at intermediate temperatures at high compared to low PC1 values, whereas no differences between PC1 values were observed at high and low temperatures (Figure 4). This leads to a pattern of stronger decrease in mammals and stronger increase in insects with increasing temperatures in high‐ than low‐quality habitats, with a constant share of insects and mammals above c. 15°C. Earthworm provisioning was higher at low PC1 values at low temperatures compared to high PC1 values, but did not differ at intermediate and high temperatures (Figure 4). Additionally, the proportion of earthworms slightly decreased with wind speed at high PC1 values, while it marginally increased at low PC1 values (Figure 4). Insect provisioning increased with wind speed at high PC1 values, while it was largely unaffected by wind speed at low PC1 values (Figure 4). No significant interaction between wind speed and PC1 was detected for mammal provisioning. Finally, the proportion of provisioned earthworms increased with rainfall across all PC1 levels but more steeply at low than at high PC1 values (Figure 4). The effects of rainfall on the proportions of insects and mammals were not modulated by PC1 (Figure 4).

## 4 Discussion

High‐quality habitats in the studied Little Owl territories were characterised by high proportions of grasslands interspersed with gardens and hedges and low proportions of arable fields, but not a high proportion of orchards. Our results showed that both the rate and biomass of prey provisioned by Little Owls increased with the area of such high‐quality foraging habitats around nests in various populations across Europe. This increase was driven by an increased provisioning of insects, accompanied by a slight decrease in mammals in high‐quality habitats. Importantly, our results also indicate that high‐quality habitats buffer against negative effects of adverse weather effects on parental provisioning, either through increased biomass at low temperatures (by increasing the proportion of small mammal prey) or through increased provisioning rates at high wind speeds (by increasing the proportion of insects). These findings underscore the crucial importance of high‐quality habitats to preserve and promote food‐limited and rapidly declining populations of Little Owls across Europe.

High‐quality habitats, particularly extensively managed grasslands, have previously been shown to be important predictors of Little Owl distribution and reproductive success in Western and Central Europe (Šálek et al. 2016; Michel et al. 2017; Apolloni et al. 2018). These habitats provide high prey availability and are therefore preferably used for foraging (Šálek, Riegert, and Křivan 2010; Mayer et al. 2021). In line with this, we found that provisioning rates and biomass provisioned to nestlings increased with high‐quality habitats, which were associated with the area of grasslands. However, the fact that high‐quality habitats were not only positively correlated with grasslands, but also with gardens and hedges suggests that the higher provisioning is also linked to greater structural and habitat diversity, resulting in landscape complementation (Michel et al. 2017). Increased habitat diversity, including edge habitats between cropped fields and non‐cropped areas (hedges) offers a high diversity of crucial food resources (Vickery et al. 2002; Fischer et al. 2009; Šálek et al. 2022), while also providing perching and roosting opportunities (see also Apolloni et al. 2018; Tschumi, Birkhofer, et al. 2020; Tschumi, Scherler, et al. 2020). Furthermore, structurally diverse habitats created through different spatio‐temporal and often small‐scale management practices can act as suitable foraging areas offering high availability and accessibility to diverse prey species throughout the entire nesting period (Whittingham et al. 2006; Schaub et al. 2010). This may be particularly true for habitat patches with short sward vegetation that facilitate hunting of ground‐dwelling prey (Šálek and Lövy 2012; Mayer et al. 2021). Finally, diverse habitat types also frequently comprise small‐scale landscape features, such as solitary trees, shrubby patches, compost piles, and manure heaps, which further increase spatial heterogeneity and provide additional foraging and perching opportunities for Little owls and other farmland bird species (Benton et al. 2003; Vickery and Arlettaz 2012; Fahrig et al. 2015; Šálek, Václav, and Sedláček 2020; Šálek, Brlík, et al. 2020; Tschumi, Birkhofer, et al. 2020; Pustkowiak et al. 2021). In contrast, we did not find a positive effect of orchard habitats on provisioning patterns. This suggests that the well‐known preference of Little Owls for orchard habitats (cf. Gottschalk et al. 2011; Šálek et al. 2016; Apolloni et al. 2018) might be due to the adequate perches, breeding cavities, and roosting sites provided by orchards rather than increased prey availability.

We also documented that the proportion of insects brought to the nest increased, while small mammals decreased with the area of high‐quality habitats around nestboxes (cf. Grüebler et al. 2018). Little Owls may preferably provision insects for two reasons: first, the energetic costs of capturing and provisioning insects may be lower than for mammals if insects are available in high numbers (Griffiths 1980). Second, small mammals are less abundant in grasslands and hedges compared to cropfields in some regions (Fischer et al. 2011), which may be particularly true for the Common Vole *Microtus arvalis* , a main mammalian prey of Little Owls (Šálek, Kreisinger, et al. 2010; Šálek, Riegert, and Křivan 2010; Sailas et al. 2024). The positive effect of habitat quality on proportion of insects, provisioning rate and provisioned biomass, taken together with the positive correlation between provisioning rate and provisioned biomass suggests that the increased provisioning of insects (smaller than average‐sized prey) in high‐quality habitats is sufficient to constitute an increase in provisioned biomass compared to low‐quality habitats. Considering this, the decline in insect populations, both in intensely managed farmlands and grasslands (Buri et al. 2016; Raven and Wagner 2021; Humbert et al. 2021), may be a major contributing factor for the population reduction of Little Owls and other birds specialising on large insects in Central and Western Europe (Hebda et al. 2019; Theux et al. 2022; Sailas et al. 2024; Birkhofer et al. 2026).

Provisioning rate and provisioned biomass peaked at intermediate values of mean daily temperature, although the peak in biomass occurred at a lower temperature than provisioning rate. For provisioning rate, this pattern likely reflects shifts in the proportion of provisioned prey types across the temperature gradient, as indicated by the multinomial model. Provisioning of insects increased with temperature, whereas provisioning of small mammals and earthworms decreased with temperature. Insects, as poikilotherms, show higher activity levels with increasing temperature (Mellanby 1939), whereas small mammals and earthworms may become more important at low temperatures as a result of decreased insect availability. Moreover, lower temperatures increase above‐ground activity of earthworms since higher ambient temperatures and drought cause them to move deeper into the ground (Onrust et al. 2019). Considering the contrasting associations of temperature with different prey types—insects increasing at higher temperatures, while small mammals and earthworms peak at lower temperatures—it is not surprising that the highest provisioning rates and provisioned biomass occurred at intermediate temperatures (13°C–15°C for provisioning rate and 10°C–12°C for biomass). However, the fact that provisioned biomass peaked at a lower temperature than provisioning rate and did not appreciably decline at low temperatures (i.e., 5°C–10°C) suggests that Little Owls may provision more biomass to balance the higher thermoregulatory costs to nestlings under cold conditions (see also Bakken et al. 2002).

Increases in average daily wind speed and total daily rainfall reduced provisioning rate and biomass, although the effect of rainfall was not statistically significant. High wind speeds can reduce the flight efficiency of birds and also alter prey accessibility. For example, high wind speeds can mask the vocalisations and sounds made by movement of small mammals through vegetation, making their capture difficult (Van Manen 2001; Geipel et al. 2019). Consistent with this, we found that provisioning of mammals decreased with increasing wind speed, whereas provisioning of insects increased. The lack of a clear effect of rainfall on provisioning rate and biomass may be explained by its contrasting effects on different prey types. Rainfall reduced insect provisioning but increased provisioning of small mammals and earthworms, corresponding with known effects of precipitation on their above‐ground activity (Macdonald 2010; Wróbel and Bogdziewicz 2015).

Importantly, we found evidence supporting our hypothesis that high‐quality habitats buffer against the negative effects of adverse weather conditions. Little Owls provisioned higher biomass to nestlings in high‐quality habitats at low temperatures, when thermoregulatory costs are expectedly higher, by provisioning a higher proportion of small mammals. We also found an indication of increased provisioning rates at high wind speeds in high‐quality habitats, although this effect was not reflected for biomass. No buffering effect of habitat quality was detected against rainfall, and overall, the effects of rainfall on provisioning rates and biomass were weak and non‐significant. However, under conditions of strong wind and rainfall, Little Owls in high‐quality habitats were less reliant on small mammals and earthworms and instead continued provisioning insects. This suggests that the high prey diversity and abundance in high‐quality habitats may be critical to allow adequate adjustments of provisioning under dynamic environmental conditions. Climatic projections indicate that temperature and precipitation extremes will increase in frequency in the future as a consequence of human‐induced climate change (Cardell et al. 2020; Zohner et al. 2020). Adverse weather conditions have been shown to negatively impact reproductive success and survival of birds (Skagen and Adams 2012; Kämpfer et al. 2022; Perrig et al. 2024) and hence may also limit population productivity in Little Owls. While several studies have demonstrated that habitat quality can buffer against the negative effects of adverse weather conditions on population growth and reproductive success in birds (Betts et al. 2018; Kujala et al. 2024), our study shows a similar buffering effect for parental provisioning. This finding therefore significantly advances our understanding of the mechanisms underlying reproduction and productivity.

Nestling age and brood size also influenced provisioning patterns. Provisioning rate and biomass increased with age at the beginning of the nestling period and declined towards fledging. Prey composition also changed across the nesting season: small mammals were provisioned more frequently in the early nestling period, insects peaked in the intermediate period, and earthworms were highest towards the end of the nestling period (see also Sailas et al. 2024). In addition, parents of large broods showed a reduced provisioning rate and biomass per chick, as well as a higher proportion of insects brought to the nest. Overall, this corresponds with known effects of nestling age and brood size on parental provisioning in birds (Senécal et al. 2021; Wojczulanis‐Jakubas et al. 2023). Particularly, the effects of age on provisioning rate and biomass, as well as on prey composition, likely reflect parents matching the energetic and physiological requirements of the brood in initial stages and reducing provisioning in later stages to encourage fledging. Yet, as Little Owl nestlings are often also provisioned outside the nestbox towards the end of the nestling period, the incomplete recording of provisioning events by cameras may additionally contribute to the observed decreased provisioning patterns towards the end of the nestling period.

## 5 Conclusions

While this study underscores the known positive effects of high‐quality habitats on provisioning rates and biomass for contrasting farmlands in Europe, it provides novel insights into the influence of weather conditions on biomass and prey composition in different habitats. For the first time, our results show that high‐quality habitats not only allow for increased food provisioning in Little Owls but also buffer against the negative effects of adverse weather conditions. This implies that the high biodiversity associated with heterogeneous and structurally diverse high‐quality habitats allows for more accurate, dynamic adjustments of foraging behaviour and prey types under changing environmental conditions. Our results therefore show that creating patches of high habitat heterogeneity and quality in intensively‐managed agricultural landscapes will not only improve breeding productivity and survival of Little Owls, but also buffer against the negative effects of climate change. Furthermore, our results also suggest that orchards per se are not essential for prey diversity and habitat quality needed by Little Owls, as often assumed. While traditional orchards may offer the structural habitat heterogeneity needed to provide the availability and accessibility of diverse prey, such conditions—along with the availability of nesting and roosting opportunities—are frequently lacking in modern orchards. In light of our findings, effective conservation measures should focus on increasing habitat heterogeneity and diversity not just within Little Owl territories, but also at the broader spatial scales. This may be achieved either through the establishment of small grassland patches and other non‐cropped habitats (e.g., hedges) or increasing representation of prey‐rich edge habitats (Benton et al. 2003; Fahrig et al. 2011; Vickery and Arlettaz 2012; Šálek et al. 2018) as well as through the diversification of management practices (e.g., partial or asynchronous mowing of grassy habitats; Cizek et al. 2012; Vickery and Arlettaz 2012; Parmentier et al. 2025). Such measures can provide high availability and accessibility of diverse prey under dynamic weather conditions throughout the year, which will be crucial for mitigating food limitation in this once‐common but now rapidly declining farmland raptor.

## Author Contributions

**S. Sangeeth Sailas:** conceptualization (lead), data curation (equal), formal analysis (lead), investigation (lead), methodology (equal), project administration (lead), software (lead), validation (lead), visualization (lead), writing – original draft (lead), writing – review and editing (equal). **Matthias Tschumi:** data curation (equal), formal analysis (supporting), investigation (equal), methodology (supporting), resources (equal), supervision (supporting), validation (supporting), writing – review and editing (equal). **Martin U. Grüebler:** conceptualization (equal), data curation (equal), formal analysis (supporting), funding acquisition (equal), investigation (equal), methodology (supporting), resources (equal), supervision (equal), validation (supporting), writing – review and editing (equal). **Filip Reipricht:** data curation (equal), investigation (equal), resources (equal). **Pascal Stroeken:** data curation (equal), investigation (equal), resources (equal). **Ronald van Harxen:** data curation (equal), investigation (equal), resources (equal). **Martin Šálek:** conceptualization (lead), data curation (equal), funding acquisition (equal), investigation (equal), methodology (equal), project administration (supporting), resources (equal), supervision (lead), writing – review and editing (equal).

## Funding

This work was supported by Schweizerischer Nationalfonds zur Förderung der Wissenschaftlichen Forschung (grant 3100A 132951/1 to B. Naef‐Daenzer and M. U. Grüebler), Karl Mayer Stiftung, Hirschmann‐Stiftung, Akademie Věd České Republiky (RVO 68081766), Akademie Věd České Republiky (Strategie AV21 Krize biodiverzity), Nature Conservation Agency of the Czech Republic, Jihočeská Univerzita v Českých Budějovicích (GAJU No. 014/2022/P, 04–072/2024/P, 047/2025/P).

## Conflicts of Interest

The authors declare no conflicts of interest.

## Supporting information


**Data S1:** ece372969‐sup‐0001‐DataS1.docx.

## Data Availability

Data are available from vogelwarte.ch Open Repository and Archive: https://zenodo.org/records/17521558 (Sailas et al. 2025).
