# Supplementary material for: Habitat‐Dependent Provisioning Patterns Are Modulated by Weather Conditions in a Rapidly Declining Farmland Raptor
Source: Ecol Evol. 2026 Jan 19;16(1):e72969. doi: 10.1002/ece3.72969 (PMC12815596; doi:10.1002/ece3.72969)
Supplement: Supplementary file 1 — Data S1: ece372969‐sup‐0001‐DataS1.docx. [file ECE3-16-e72969-s001.docx]

## **Supporting Information**

**Figure S1**: Map of study areas showing the locations of studied nestboxes in individual countries.


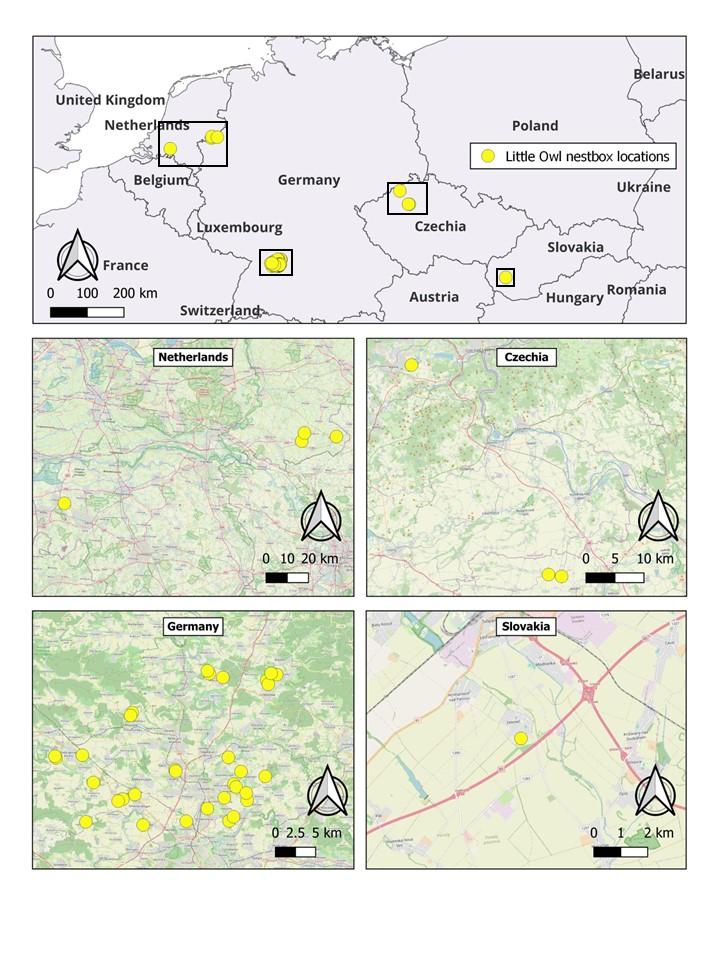


**Table S1**: Loadings of the first two Principal Component (PC) axes created using habitat variables.

|  | **Comp.1** | **Comp.2** |
| --- | --- | --- |
| **Arable field area** | 0.570 | 0.355 |
| **Grassland area** | -0.537 | 0.118 |
| **Orchard area** |  | -0.855 |
| **Hedges area** | -0.433 |  |
| **Garden area** | -0.443 | 0.353 |

**Figure S2**: Biplot showing the quality of representation (cos2) of habitat variables used in the Principal Component Analysis (PCA).


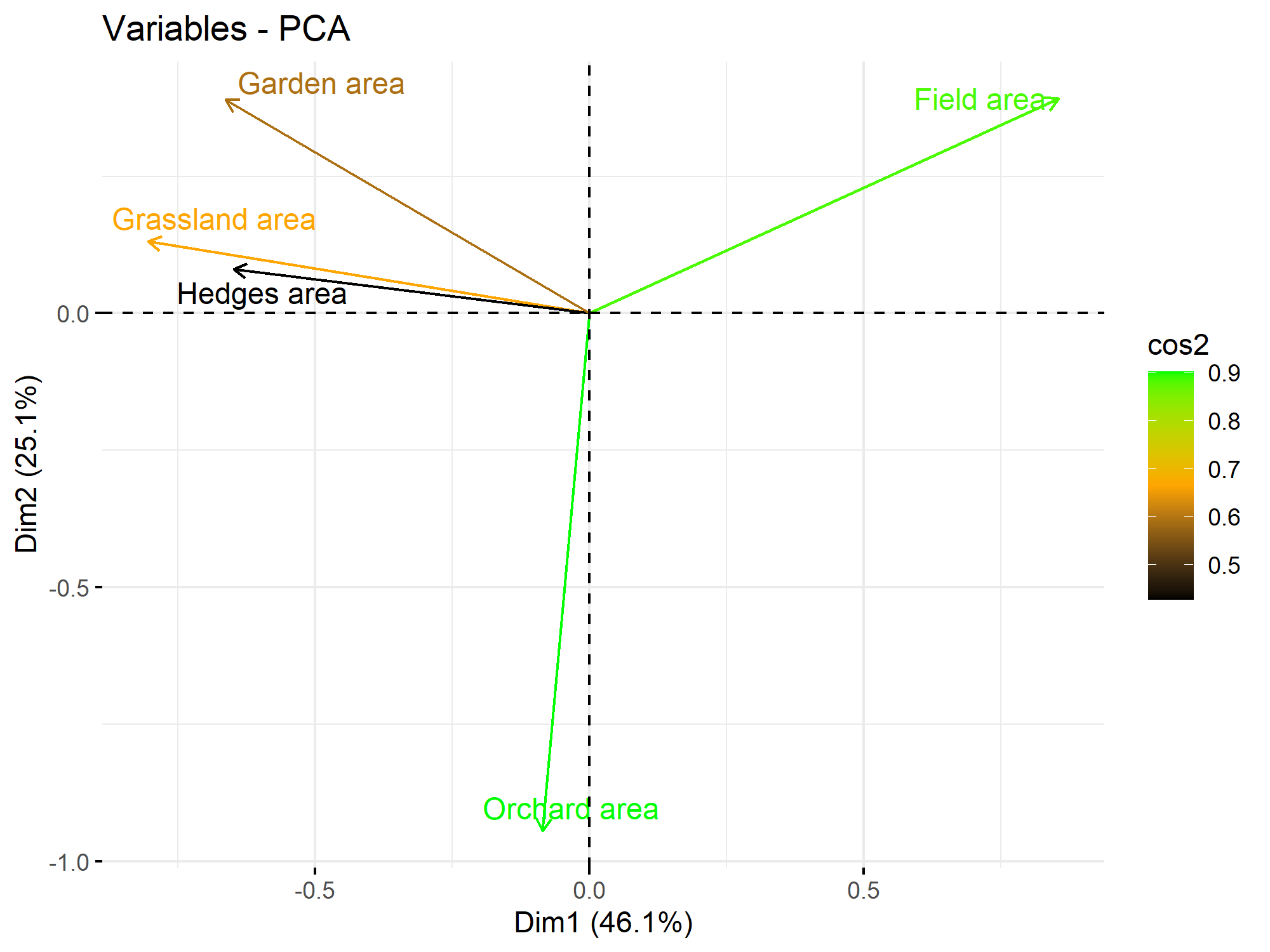


**Figure S3:**  Relationship between provisioning rate (per day and per chick) and provisioned biomass (per day and per chick). The blue line indicates the fitted regression line and the red, dashed line represents the expected relationship between provisioning rate and provisioned biomass, if biomass per provisioned prey item remains constant at the mean biomass value across provisioning rate values.


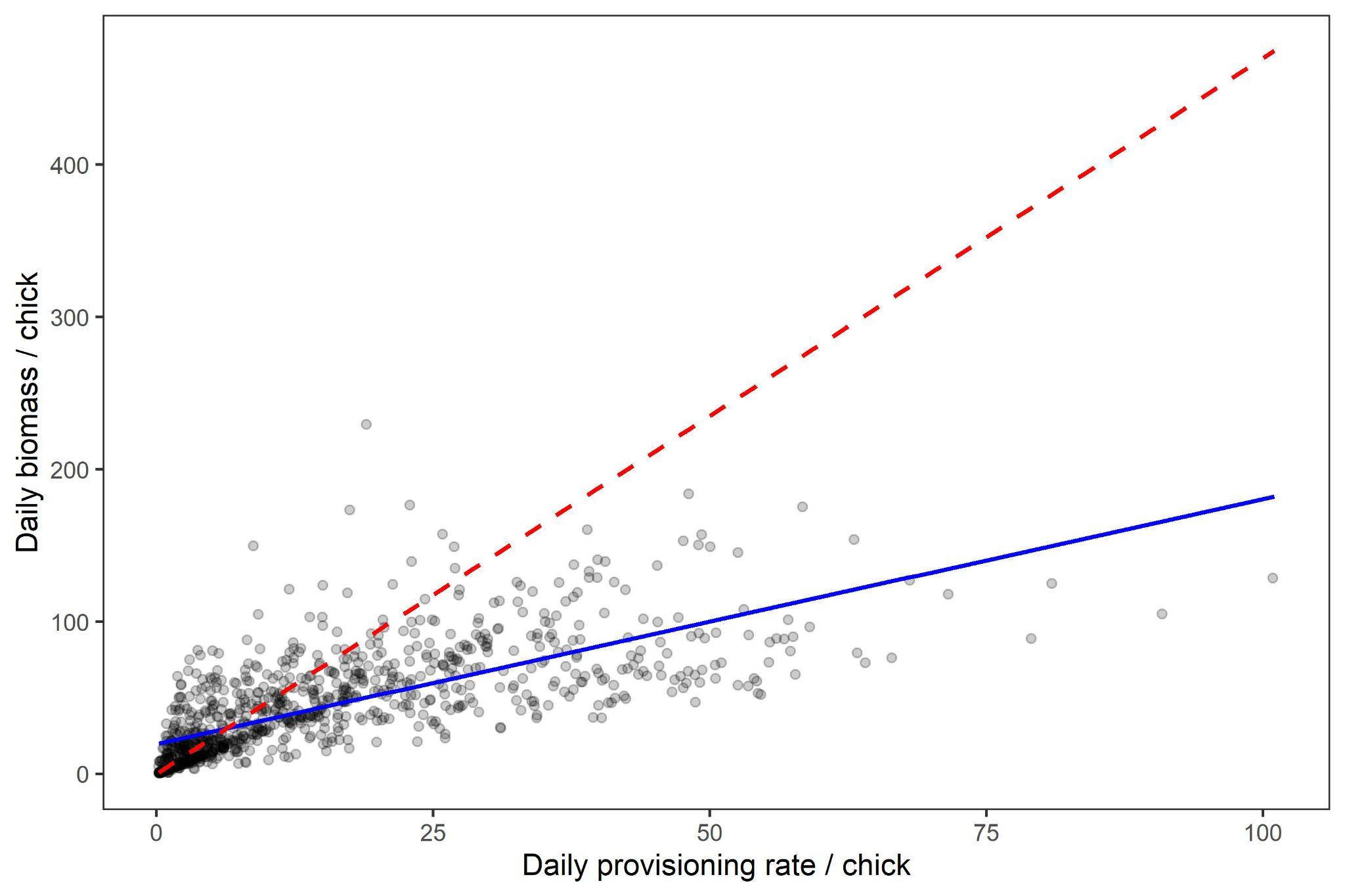


**Table S2**: Results from the LMM assessing the effect of *country*, along with other predictors on provisioning rate in breeding Little Owls, without half-days (days whether nest monitoring took place only for half of the day). Czechia and Slovakia (considered as one factor level) were taken as the reference level. The dataset was filtered to include only the nests with PC1 values overlapping across the four countries, so that the assessment of the country effect on provisioning rate would not be confounded by the effect of PC1. Provisioning rate was cubic-root transformed and predictors were centred and scaled before their inclusion in the models.

| **Provisioning rate** | | | |
| --- | --- | --- | --- |
| ***Predictors*** | *Estimates* | *SE* | *p* |
| **(Intercept)** | 1.68 | 0.57 | **0.004** |
| **age** | 0.36 | 0.05 | **<0.001** |
| **age^2** | -0.22 | 0.04 | **<0.001** |
| **brood size** | 0.32 | 0.20 | 0.122 |
| **country2 [Germany]** | -0.31 | 0.44 | 0.483 |
| **country2 [Netherlands]** | 0.60 | 0.59 | 0.309 |
| **PC1** | 0.34 | 0.51 | 0.502 |
| **PC2** | -0.09 | 0.13 | 0.479 |
| **temp** | -0.18 | 0.05 | **<0.001** |
| **temp^2** | -0.08 | 0.03 | **0.011** |
| **rain** | 0.01 | 0.04 | 0.855 |
| **wind** | 0.01 | 0.08 | 0.860 |

**Table S3**: Results from the LMM assessing the association of predictors with provisioning rate in breeding Little Owls, without half-days (nest monitoring took place only for half of the day). Provisioning rate was cubic-root transformed and predictors were centred and scaled before their inclusion in the models.

|  | **Provisioning rate** | | |
| --- | --- | --- | --- |
| ***Predictors*** | *Estimates* | *SE* | *p* |
| **(Intercept)** | 2.14 | 0.11 | **<0.001** |
| **age** | 0.19 | 0.02 | **<0.001** |
| **age^2** | -0.29 | 0.01 | **<0.001** |
| **brood size** | -0.06 | 0.05 | 0.199 |
| **PC1** | 0.22 | 0.10 | **0.025** |
| **PC2** | -0.13 | 0.09 | 0.117 |
| **temp** | -0.05 | 0.02 | **0.010** |
| **temp^2** | -0.05 | 0.01 | **<0.001** |
| **rain** | -0.02 | 0.02 | 0.112 |
| **wind** | -0.04 | 0.02 | **0.034** |
| **PC1 × temp** | -0.02 | 0.01 | 0.196 |
| **PC1 × temp^2** | -0.05 | 0.01 | **<0.001** |
| **PC1 × wind** | 0.03 | 0.02 | 0.194 |

**Table S4**: Results from the multinomial model assessing the association of predictors with composition of provisioned prey in breeding Little Owls. ‘Insect’ was considered as the reference category. *p* values of significant associations are given in bold.

| **Mammal vs insect:** | | | | |
| --- | --- | --- | --- | --- |
|  | Estimate | SE | z value | *p* |
| **(Intercept)** | -1.70 | 0.23 | -7.30 | **<0.001** |
| **age** | -0.14 | 0.03 | -4.03 | **<0.001** |
| **age^2** | 0.39 | 0.03 | 13.33 | **<0.001** |
| **brood size** | -0.45 | 0.10 | -4.65 | **<0.001** |
| **PC1** | -0.59 | 0.22 | -2.64 | **0.008** |
| **PC2** | 0.22 | 0.21 | 1.08 | 0.280 |
| **temp** | -0.15 | 0.04 | -3.97 | **<0.001** |
| **temp^2** | 0.04 | 0.02 | 1.82 | 0.069 |
| **rain** | 0.16 | 0.03 | 4.87 | **<0.001** |
| **wind** | -0.08 | 0.04 | -1.95 | 0.052 |
| **PC1:temp** | 0.03 | 0.04 | 0.91 | 0.362 |
| **PC1:temp^2** | 0.09 | 0.03 | 3.52 | **<0.001** |
| **PC1:rain** | -0.01 | 0.03 | -0.16 | 0.876 |
| **PC1:wind** | -0.08 | 0.05 | -1.81 | 0.070 |
|  |  |  |  |  |
|  |  |  |  |  |
|  |  |  |  |  |
| **Earthworm vs insect:** | | | | |
|  | Estimate | Std. Error | z value | Pr(>\|z\|) |
| **(Intercept)** | -0.63 | 0.29 | -2.20 | **0.028** |
| **age** | 1.00 | 0.03 | 37.72 | **<0.001** |
| **age^2** | -0.04 | 0.03 | -1.78 | 0.076 |
| **brood size** | -0.30 | 0.08 | -3.94 | **<0.001** |
| **PC1** | -0.28 | 0.27 | -1.03 | 0.302 |
| **PC2** | 0.23 | 0.25 | 0.90 | 0.366 |
| **temp** | -0.38 | 0.03 | -14.21 | **<0.001** |
| **I(temp^2)** | -0.02 | 0.02 | -0.93 | 0.353 |
| **rain** | 0.25 | 0.02 | 10.59 | **<0.001** |
| **wind** | -0.01 | 0.03 | -0.38 | 0.707 |
| **PC1:temp** | -0.01 | 0.02 | -0.30 | 0.767 |
| **PC1:temp^2** | -0.05 | 0.02 | -2.83 | **0.005** |
| **PC1:rain** | -0.07 | 0.02 | -4.48 | **<0.001** |
| **PC1:wind** | -0.06 | 0.03 | -1.96 | **0.050** |
